# Inflammation and depression: Research designs to better understand the mechanistic relationships between depression, inflammation, cognitive dysfunction, and their shared risk factors

**DOI:** 10.1016/j.bbih.2021.100278

**Published:** 2021-05-27

**Authors:** Naoise Mac Giollabhui

**Affiliations:** Department of Psychology, Temple University, Weiss Hall, 1701 N. 13th St, Philadelphia, PA, 19122, USA

**Keywords:** Inflammation, Depression, Cognitive functioning, Intervention, Treatment, Exercise, Diet, Adiposity, BMI, C-reactive protein, Interleukin-6

## Abstract

There is convergent evidence that the immune system is dysregulated in some depressed individuals. A psychoneuroimmunology-based understanding of depression is advancing rapidly; however, a question of fundamental importance is poorly understood: does inflammation play a causal role in the etiology of depression or are elevated inflammatory biomarkers a downstream effect of depressive behaviors? Although longitudinal studies suggest that the relationship between depression and inflammation is characterized by complex bidirectional associations, existing prospective, longitudinal research designs are poorly equipped to investigate the dynamic interplay of depression and inflammation that unfolds over a relatively short time period. In addition, the precise role played by multiple, shared, and overlapping risk factors (e.g., diet, adiposity, stress, sleep dysregulation) in the etiology of depression and a pro-inflammatory phenotype (or both) is poorly understood. In this manuscript, I highlight the benefits of research designs that (i) manipulate constructs of interest (depression/inflammation) using intervention or treatment designs and (ii) use intensive sampling approaches with an ultimate goal of better understanding the temporal sequence and causal relationships of depression, inflammation, cognitive dysfunction, and their shared risk factors. For instance, are improved depressive symptoms a downstream effect of changes in inflammatory activity caused by increases in exercise or, alternatively, are changes in inflammatory activity and depression sequelae of improvements in sleep quality caused by increases in exercise? Potential benefits of these research designs are discussed in terms of their contribution to a better understanding of the etiology of depression and a pro-inflammatory phenotype, their relevance to structural health inequalities, and better characterizing the heterogeneous clinical presentation of depression, particularly relating to the etiology of cognitive dysfunction in depression.

## Highlights

• Evidence that inflammation plays a causal role in the etiology of depression (as well as the reverse) is reviewed.
• The causal relationship between depression and inflammation is unclear due to multiple, shared, and overlapping risk factors.
• Approaches to investigate the causal relationships of depression, inflammation, and cognitive dysfunction are discussed.

## 1 Introduction

**Fig. 1:**
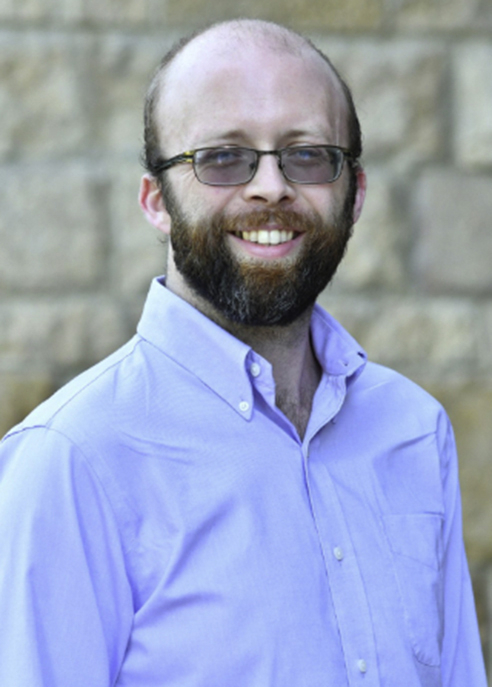
**Naoise Mac Giollabhui**. Naoise Mac Giollabhui's research seeks to understand why cognitive functioning is disrupted (e.g., attention, memory, and executive functioning) during a depressive episode and why these cognitive difficulties persist in remitted depression. In particular, his work focuses on the role that the immune system plays in the etiology of depression and cognitive dysfunction. Two important themes have emerged from his published work. First, inflammation may be associated with worse cognitive functioning, particularly worse executive functioning, in both depressed and non-depressed individuals. Second, the relationship between inflammation and depression is characterized by complex, bidirectional associations and understanding their causal relationship requires a clearer understanding of the role played by multiple risk factors that inflammation and depression share, such as low socioeconomic-status, stress, adiposity and diet. Naoise's current work has two primary areas of investigation. The first is to better understand how immune functioning relates to specific dimensions of cognitive functioning in both acute and remitted depression. The second is to disentangle the role played by multiple, shared, and overlapping risk factors (e.g., diet, adiposity, stress) in the etiology of depression, a pro-inflammatory phenotype, and cognitive dysfunction. Naoise completed his undergraduate education in psychology at University College Dublin and began his clinical psychology Ph.D in 2015 working with Dr. Lauren Alloy in the Mood and Cognition Lab at Temple University. He completed his clinical training at Temple University's Psychological Services Center and the Children's Hospital of Philadelphia's Neuropsychology and Assessment Service, and he is looking forward to starting his clinical internship at Massachusetts General Hospital's Cognitive Behavioral Therapy track in July 2021. His research has been supported by a number of awards and grants, including a National Institute of Mental Health National Research Service Award and an American Psychological Foundation Visionary Grant.

**Fig. 2:**
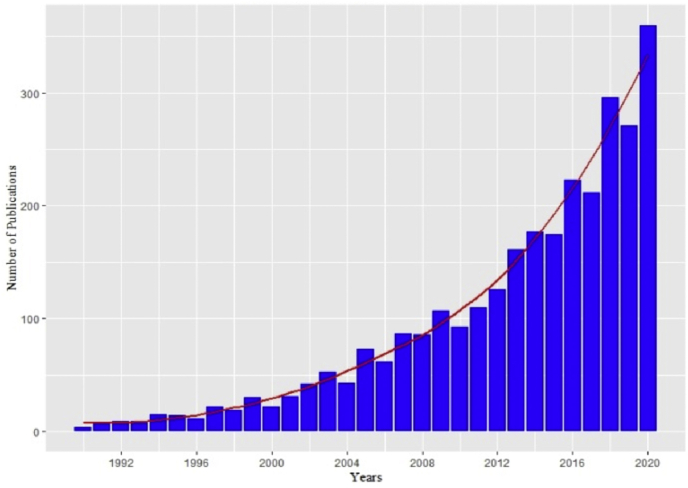
Studies of depression and inflammation published between 1990 and 2020.

Depression afflicts an estimated 300 million people around the world (Herrman et al., 2019). Not only is depression highly prevalent, but it typically emerges early in life, follows a recurrent course, and is difficult to treat (Burcusa and Iacono, 2007; Herrman et al., 2019; Kessler et al., 2003; Rush et al., 2006) – characteristics that help explain why it is the most burdensome of psychological disorders (Erskine et al., 2015). The cardinal symptoms of depression are low mood and anhedonia, but it often is accompanied by a diverse set of symptoms, including dysregulated sleep, appetite, and/or cognition (American Psychiatric Association, 2013); in fact, the specific relationship between depression and cognitive functioning is a question that I am particularly interested in - see Fig. 1. Depression's heterogeneous presentation suggests that different subtypes of depression exist and developing personalized treatment strategies based on subtype presentation may lead to more efficacious interventions (Kunugi et al., 2015). Since a dysregulated immune system first was hypothesized to play a causal role in the etiology of depression in the early 1990s (Maes et al., 1992; Smith, 1991), the number of studies investigating the relationship between depression and inflammation has increased dramatically – see Fig. 2 – culminating in more than 350 articles published in 2020.^1^

Over the last 30 years, psychoneuroimmunology research on depression has made considerable methodological, empirical, and conceptual advances. Reliable experimental research designs have emerged to examine the acute effects of inflammation (Lasselin et al., 2020; Schedlowski et al., 2014). There is growing appreciation of multiple factors that influence measurement of circulating inflammatory biomarkers (O'Connor et al., 2009), the genetic basis of inflammation in depression (Khandaker et al., 2019), and an inflammatory phenotype of depression characterized by somatic/energy-related symptoms (Milaneschi et al., 2020). There is compelling evidence from observational studies that inflammatory biomarkers are elevated in clinically depressed individuals (Dowlati et al., 2010; Haapakoski et al., 2015) and clinical trials continue to examine the efficacy of anti-inflammatory medications (Nettis et al., 2021). Finally, elegant theories of depression and inflammation are being elaborated (Slavich, 2020; Slavich and Irwin, 2014) and there is a better understanding of the mechanisms by which peripheral inflammation affects the central nervous system (Miller and Raison, 2016). Despite this growth in knowledge, the answer to a question of fundamental importance is unknown: do inflammatory processes play a causal role in the etiology of depression or are elevated inflammatory biomarkers caused by depressogenic behaviors? Excellent reviews highlight multiple pathways by which inflammation may lead to depression (e.g., genetics, early childhood adversity, stress) and by which depression may lead to inflammation (e.g., sedentary behavior, diet, substance use) (Berk et al., 2013; Kiecolt-Glaser et al., 2015; Raison et al., 2006); however, existing research designs (primarily prospective, longitudinal studies) are poorly equipped to answer this question and empirical data are equivocal (Mac Giollabhui et al., 2020b). In this paper, I intend to, first, review research exploring the causal relationship between depression and inflammation. Second, I will identify the methodological challenges we need to overcome to better understand the causal relationship between depression and inflammation. Third, I will propose approaches that may help the field advance our understanding of this topic. Finally, I will consider the benefits and challenges of implementing such approaches, particularly as they relate to cognitive dysfunction in depression.

## 2 Is the immune system dysregulated in depression?

Sickness behaviors (e.g., anhedonia, fatigue) that occur when the immune system responds to a pathogen (e.g., influenza virus) overlap considerably with depressive behaviors and this observation has spurred interest in the role of the immune system in depression (Dantzer et al., 2008; Hart, 1988). There is now convergent evidence that the innate immune system is dysregulated in depression. First, depression is frequently comorbid across a range of medical conditions involving dysregulation of the immune system (e.g., rheumatoid arthritis) (Dregan et al., 2019; Slavich and Irwin, 2014). Second, depressive symptoms reliably follow induction of an innate immune response in both treatment (e.g., interferon-α) (Udina et al., 2012) and experimental studies (e.g., administration of an endotoxin) (Dantzer et al., 2008; Schedlowski et al., 2014). Third, observational data indicate that peripheral inflammatory biomarkers, including interleukin-6 (IL-6), and C-reactive protein (CRP), are consistently elevated in depression (Dowlati et al., 2010; Haapakoski et al., 2015; Howren et al., 2009; Kohler et al., 2017). Fourth, a recent Mendelian randomization study highlights accumulating evidence, albeit inconsistent at times (Barnes et al., 2017), that genetically determined levels of inflammation play a causal role in the onset of depression (Khandaker et al., 2019). Finally, there also is evidence that some depressed individuals may benefit from treatment with anti-inflammatory agents (Husain et al., 2017; Kohler et al., 2016; Rosenblat and McIntyre, 2018). These studies provide evidence that inflammation may play a causal role in depression, although probably only for the subset of depressed individuals (≈25%) who exhibit elevated inflammatory biomarkers (CRP≥3 ​mg/L) (Osimo et al., 2019). However, there also is conflicting evidence that inflammation, rather than being a cause of depression, is a consequence of the unhealthy behaviors that frequently accompany depression.

## 3 Does depression lead to elevated inflammation?

As reviewed, there is considerable evidence that inflammation plays a causal role in the onset of depression for some individuals; however, it is equally plausible that elevated inflammatory biomarkers are a downstream effect of depressogenic behaviors (e.g., sedentary behavior, substance use, poor sleep, and a high-fat, high-sugar diet) (Berk et al., 2013). Indeed, conflicting evidence as to the direction of the association of depression and inflammatory biomarkers is highlighted in a recent systematic review and meta-analysis (Mac Giollabhui et al., 2020b). Mac Giollabhui et al. examined the prospective associations of inflammatory biomarkers and depression in population-representative, longitudinal studies and reported evidence of bidirectional associations for two commonly used inflammatory biomarkers (CRP and IL-6). Importantly, the magnitude of the observed associations often was attenuated substantially after adjusting for demographic (e.g., sex), cognitive (e.g., stress), and biological (e.g., adiposity, triglycerides) factors. In fact, in the case of baseline depression predicting future CRP, the number of studies reporting a significant association dropped from 65% to 6% following adjusting for covariates and, in the case of baseline CRP predicting future depression, the number of significant associations dropped from 52% to 20% after adjusting for covariates. Although it is well known that levels of circulating peripheral inflammatory biomarkers are influenced by a wide range of sampling (e.g., fasting status), demographic (e.g., sex, race), cognitive (e.g., stress), behavioral (sedentary behavior, exercise, diet), and clinical characteristics of depression (e.g., medication use) (Mac Giollabhui et al., 2020a, Mac Giollabhui et al., 2020b; O'Connor et al., 2009), little is known about which of these factors represent “noise” in the data (e.g., diurnal variation in inflammatory biomarkers due to differences in time of blood draw) and which are causally linked to depression via inflammation. For instance, adipose tissue is responsible for approximately 30% of circulating IL-6, which directly stimulates CRP (Mohamed-Ali et al., 1997), and also is linked to depression (Quek et al., 2017); thus, does examining the relationship of CRP and depression adjusted for adiposity misrepresent the potentially causal relationship linking adiposity and depression via inflammation (Berk et al., 2013)? Importantly, the scope of the problem extends far beyond adiposity to encompass a wide range of related factors, such as socioeconomic status (SES) and diet, which are both associated with adiposity, inflammatory physiology, and depression (Booth et al., 2017; Calder et al., 2011; Malik et al., 2009; Muscatell et al., 2018; Shivappa et al., 2017)? When considered as a whole, these data provide strong empirical support for bidirectional associations of depression and inflammation and identify risk factors that may play a role in the emergence of both a pro-inflammatory phenotype and depression. In fact, one of the greatest challenges researchers in this field face is disentangling the many variables that are independently associated with both depression and inflammation.

## 4 Which risk factors contribute to inflammation in depression and how should we conceptualize them?

Multiple risk factors, beyond adiposity, diet, and SES, may be associated with depression and inflammation. Stress frequently precedes, and generally is believed to play a causal role in, the onset of depression (Hammen, 2005) and is associated with elevated levels of inflammatory activity (Rohleder, 2019). Indeed, recent theories offer a compelling explanation of the interplay of stress and inflammation in the etiology of depression (Slavich, 2020; Slavich and Irwin, 2014). Similarly, persistent sleep difficulties are implicated in the onset of both depression and inflammation (Lopresti et al., 2014). Higher levels of exercise are associated with lower incidence of depression – in fact, exercise interventions compare favorably to pharmacological intervention as a first-line intervention for individuals with mild-to-moderate depression (Carek et al., 2011) – and exercise also is associated with long-term anti-inflammatory effects (Lopresti et al., 2013). Substance use, such as smoking, similarly is linked closely with depression and inflammation (Gonçalves et al., 2011; Taylor et al., 2014). The importance of these risk factors in the etiology and maintenance of depression is outlined in multiple systematic reviews identifying smoking cessation, increased exercise, improved diet/nutrition and sleep as effective interventions for depression (Ballesio et al., 2018; Jacka et al., 2017; Kvam et al., 2016; Lopresti et al., 2013; Schuch et al., 2016; Taylor et al., 2014).

Importantly, there is considerable evidence that, in addition to being independently linked with depression and inflammation, many of these risk factors overlap considerably with one another; in other words, individuals of low SES are more likely to have a poor diet, to experience stress, to exercise less, and to sleep less well (Gold et al., 2020; O'Connor et al., 2009; Slavich, 2020). That these modifiable risk factors are implicated in both depression and inflammation and overlap considerably with one another is clear; what remains unknown, however, is whether they are causally linked with inflammation and/or depression. For instance, is inflammation prospectively associated with depression only because it is a risk marker for poor diet, which is causally linked to depression? Or, is it that depression is caused by elevated inflammatory physiology, which is caused by dysregulated sleep? In order to comprehensively understand the causal relationship of inflammation and depression, further work is needed to characterize how multiple, overlapping risk factors contribute to inflammation in depression (or the reverse).

## 5 What are methodological challenges we need to overcome to understand how multiple, overlapping risk factors contribute to inflammation in depression?

In the systematic review and meta-analysis of the prospective associations of inflammatory biomarkers and depression, Mac Giollabhui et al. (2020) identified major challenges the field faces when trying to understand the causal relationships between inflammation and depression and the contributory role played by shared risk factors. First, the timing of assessments in most observational studies varied considerably and follow-up assessments typically occur 4 years after baseline assessment. Long intervals between assessments make it difficult to disentangle the bidirectional associations that characterize the relationship between depression and inflammation. Moreover, that assessments are repeated years apart mean that observational studies rarely possess the temporal resolution to examine the potentially mediating pathways by which inflammation and depression are linked. Second, 75% of studies reviewed were carried out in adult or elderly samples (mean age ​= ​51 years). Consequently, understanding the temporal sequence of depression and inflammation is challenging because the downstream effects of depressogenic behaviors on circulating inflammatory biomarkers may already be embedded at the baseline assessment in older samples. These methodological challenges limit out conceptual understanding of the causal relationship between depression and inflammation.

## 6 What types of research designs can overcome these methodological challenges?

Greater utilization of research designs with a number of characteristics may be useful in overcoming these methodological challenges. First, in order to parse apart the temporal relationship between depression, inflammation, and their shared risk factors (e.g., diet, substance use, dysregulated sleep), research designs capable of repeatedly assessing multiple constructs with fine temporal resolution (e.g., ecological momentary assessment) will be vital in characterizing the temporal sequence by which change occurs (e.g., does increases in inflammatory physiology lead to depressed mood via sleep dysregulation or is it the reverse). Second, repeated assessment methodologies will be most usefully deployed in research designs where inflammation/depression is being manipulated (e.g., interferon-α treatment, exercise/diet intervention) in order that change predictably occurs and hence, can be measured within a period of intensive sampling. Third, greater focus on youth is needed to definitively identify which variable(s) exert an initial effect on our variables of interest, particularly given the preponderance of studies on inflammation and depression in older adults when it is difficult to parse apart whether depression or inflammation developed first.

**Fig. 3:**
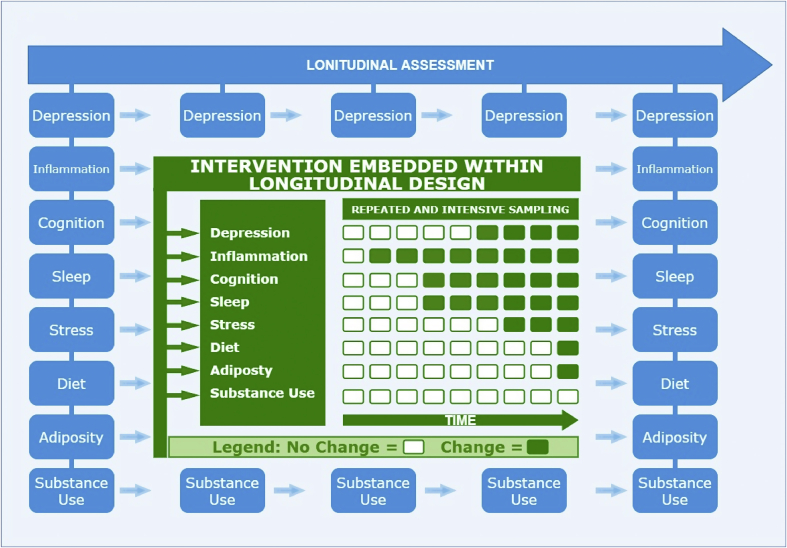
Integrating intervention and intensive sampling methodologies within longitudinal observational studies.

Embedding the above three approaches within longitudinal studies may be particularly useful at evaluating whether intervention leads to enduring changes in depression/inflammation. In an ideal world, these approaches would be incorporated within a large-scale, multi-site prospective longitudinal study of adolescents that includes both intervention and ecological momentary assessment. For instance, following the recruitment of youth aged 12–13 years (who are unlikely to have developed depression or exhibit elevated inflammatory physiology) and establishing longitudinal trajectories of depression, inflammation, and their shared risk factors using regular prospective assessment, participants could receive an intervention that targets depression and/or inflammatory physiology. During the course of the intervention, ecological momentary assessment would be critical for establishing the temporal order by which change in depression and inflammation occurs as well as the role of potential mediators (e.g., improved sleep) – see Fig. 3 for a visual depiction. It is important to note that, although comparable research designs have been implemented (e.g., Environmental Influences on Child Health Outcomes program), major challenges exist to fund and conduct such studies, particularly: the large sample size required to reliably detect modest effect sizes; the heterogeneity in pathways linking depression and inflammation across individuals; and the practical challenges of attrition/participant adherence to intervention.

Fig. 3 provides a visual depiction of embedding an intervention-based study and a repeated, intensive sampling methodology within a prospective, longitudinal study. Blue graphics represent repeated prospective assessment occurring within a cohort study design. In the center of the figure, the same constructs also are being repeatedly measured using a repeated and intensive sampling methodology following intervention (depicted in green (in the web version)). Intervention is designed to induce change in constructs of interest, particularly inflammation and depression, so that the temporal sequence of change in inflammatory physiology, depression and their shared risk factors can be characterized. Green (in the web version) and white boxes in the center of the figure represent repeated assessments of constructs of interest within the intervention using an intensive sampling methodology; a potential pattern of change is represented using different colored boxes where green (in the web version) indicates change in a given variable and white represents no change (see legend). As depicted in the figure, longitudinal assessment would ideally continue following intervention period.

## 7 What are the benefits of incorporating these approaches?

First, the proposed research approaches may help address limitations of observational studies by observing the temporal order in which change in depression and inflammation occurs following manipulation. Observational studies, to date, have not possessed sufficient temporal acuity to truly observe the temporal sequence by which depression and inflammation change over time. Second, implementing an intensive sampling methodology following intervention/treatment creates an opportunity to better understand how shared and overlapping risk factors are associated with inflammation and depression. Establishing which risk factors precede/follow changes in inflammation/depression may provide evidence as to their causal relationships. For instance, should sleep dysregulation follow inflammation and predict future depression, this might indicate a causal chain linking inflammation and depression, including a potential mechanism. Third, many of the risk factors discussed in this paper are both modifiable and implicated in structural health disparities that contribute to worse physical and mental outcomes in marginalized communities; however, it is largely unknown which of these modifiable risk factor should be the target of intervention. In particular, identifying modifiable risk factors that are causally related to depression and elevated inflammatory physiology could be effective targets for intervention and, thus, of relevance to public policy. Finally, inflammation in depression may be particularly associated with somatic symptoms (e.g., fatigue) and/or cognitive dysfunction (e.g., difficulties in concentration/decision-making) and consequently a deeper understanding of inflammation in depression may contribute to a better understanding of depression's heterogeneous clinical presentation (Chu et al., 2019; Fried et al., 2019; Moriarity et al., 2020). In particular, such an approach may inform our understanding of cognitive dysfunction in depression, which is of particular importance given the clinical heterogeneity of depression and that cognitive dysfunction is differentially associated with worse functional impairment in depression (Jaeger et al., 2006; Whiteford et al., 2013; Woo et al., 2016).

## 8 What can examining the relationships between inflammation, depression, and shared risk factors tell us about cognitive dysfunction in depression?

Not only is there a subset of depressed individuals who exhibit elevated inflammatory biomarkers, but a subset of depressed individuals also experience cognitive dysfunction. These difficulties are reflected in the diagnostic criteria for depression (difficulties in concentration/decision-making) (American Psychiatric Association, 2013) and in behavioral assessments of cognition, particularly episodic memory, attention, and executive functions (Rock et al., 2014). Cognitive dysfunction is observable at first onset of depression (Ahern and Semkovska, 2017; Lee et al., 2012) and persists for many individuals when depression is in remission (Rock et al., 2014; Semkovska et al., 2019). The cause of cognitive dysfunction in depression is unknown, although inflammation is suspected to play a causal role (Carvalho et al., 2014) and has been linked with impaired cognition in both medical (Crisan et al., 2014; Huang et al., 2016; Li et al., 2014) and healthy samples (Baune et al., 2008; Cullen et al., 2017; Mac Giollabhui et al., 2021; Noble et al., 2010; Reichenberg et al., 2001; Singh-Manoux et al., 2014). Significantly, inflammation also has been linked with worse cognitive functioning in both depressed individuals and non-depressed controls (Chang et al., 2012; Goldsmith et al., 2016; Grassi-Oliveira et al., 2011; Krogh et al., 2014), suggesting that the link between inflammation and cognitive functioning is unlikely to be unique to depression. The shared risk factors (i.e., substance use, sedentary behavior, adiposity, inflammation, and exposure to life stress) discussed in this review are associated with depression and elevated inflammatory biomarkers as well as worse cognitive functioning (Esteban-Cornejo et al., 2015; Falck et al., 2017; Liang et al., 2014; Lundqvist, 2005; Lupien et al., 2009; Nooyens et al., 2008; Reichenberg et al., 2001). Indeed, work by our group has demonstrated that adiposity, stress exposure, early life adversity, and sedentary behavior are all linked to cognitive dysfunction in youth and present in depression (Mac Giollabhui et al., 2019, Giollabhui et al., 2021), with one study identifying interleukin-6 as the mediator linking adiposity with executive dysfunction (Mac Giollabhui et al., 2020c). Thus, the proposed research approach easily could be extended to address a topic of considerable theoretical and practical importance: why do depressed individuals exhibit difficulties in cognitive functioning that often persist when depression is in remission?

## 9 Conclusion

Psychoneuroimmunology research has contributed substantively to our understanding of depression; however, our conceptual understanding of the bidirectional associations of depression and inflammatory biomarkers is limited, particularly as it relates to the role of shared risk factors. Prospective, longitudinal designs using protracted follow-up assessments are poorly equipped to characterize the complex prospective associations of depression, inflammation, and their shared risk factors. Instead, intervention studies using intensive sampling methodologies may be well-positioned to examine the sequence of change occurring across multiple variables of interest within a compressed timeframe, which may deepen our understanding of the causal relationship between depression and inflammation. Such a line of research is of particular importance because many of these risk factors speak to structural disparities in health and may inform both treatment and prevention strategies as well as particularly debilitating features of depression, such as cognitive dysfunction.

## Declaration of competing interest

The author has no disclosures or conflicts of interest to report.

## Footnotes

1 Fig. 2 is based on a search of three databases (MEDLINE, APA PsycArticles, and APA PsycInfo) where the words “depress∗ OR mood OR affect” and “inflamm∗ OR interleukin OR IL-1 OR IL-6 OR Tumor Necrosis Factor OR TNF OR C-Reactive OR CRP OR cytokine” appeared in the title of humans studies.
